# Overweight and Obesity in Patients with Congenital Heart Disease: A Systematic Review

**DOI:** 10.3390/ijerph18189931

**Published:** 2021-09-21

**Authors:** Laura Willinger, Leon Brudy, Michael Meyer, Renate Oberhoffer-Fritz, Peter Ewert, Jan Müller

**Affiliations:** 1 Department of Congenital Heart Disease and Pediatric Cardiology, Deutsches Herzzentrum München, Technische Universität München, 80636 Munich, Germany; leon.brudy@tum.de (L.B.); michael.meyer@tum.de (M.M.); renate.oberhoffer@tum.de (R.O.-F.); ewert@dhm.mhn.de (P.E.); j.mueller@tum.de (J.M.); 2 Institute of Preventive Pediatrics, Technische Universität München, 80992 Munich, Germany; 3 DZHK (German Centre for Cardiovascular Research), Partner Site Munich Heart Alliance, 80636 Munich, Germany

**Keywords:** congenital heart disease, overweight, obesity, body constitution

## Abstract

Background: Overweight and obesity have become a major public health concern in recent decades, particularly in patients with chronic health conditions like congenital heart disease (CHD). This systematic review elaborates on the prevalence and the longitudinal development of overweight and obesity in children and adults with CHD. Methods: A systematic literature search was conducted in PubMed, Cochrane, and Scopus from January 2010 to December 2020 on overweight and obesity prevalence in children and adults with CHD. Results: Of 30 included studies, 15 studies evaluated 5680 pediatric patients with CHD, 9 studies evaluated 6657 adults with CHD (ACHD) and 6 studies examined 9273 both pediatric patients and ACHD. Fifteen studies received the quality rating “good”, nine studies “fair”, and six studies “poor”. In children with CHD, overweight prevalence was between 9.5–31.5%, and obesity prevalence was between 9.5–26%; in ACHD, overweight prevalence was between 22–53%, and obesity was between 7–26%. The prevalence of overweight and obesity was thereby similar to the general population. Overweight and obesity have been shown to increase with age. Conclusion: The prevalence of overweight and obesity in children and adults with CHD is similar to the general population, demonstrating that the growing obesity pandemic is also affecting the CHD population.

## 1. Introduction

Overweight and obesity have become major public health concerns in recent decades [1] and are referred to as a global pandemic [2]. The prevalence of childhood obesity has more than doubled in the last three decades [3], which is particularly concerning as childhood obesity tracks into adulthood [4]. Overweight and obesity, in particular, are independent risk factors for cardiovascular diseases and are associated with increased risk of morbidity and mortality in the general population [5]. As the majority of children with congenital heart disease (CHD) nowadays survive into adulthood [6], environmental and behavioral health risk factors are also of concern in this patient cohort [7,8]. As patients with pre-existing heart conditions are prone to cardiovascular events [9] due to underlying anatomic and functional abnormalities [10], overweight and obesity need to be considered here too.

At first glance, one would assume that patients with CHD are at increased risk for overweight and obesity because, as cardiac patients, physical activity may be reduced due to exercise restriction or overprotection, and patients participate less in leisure and competitive sports [11]. On the other hand, surgeries, hospitalization, and rehabilitation early in life affect the development of children with CHD, which can be linked to underweight and malnutrition [12]. Additionally, many CHD patients have a very strong health awareness and maintain a mindful lifestyle, including a healthy body weight [13].

So far, only limited and conflicting data exist on the prevalence of overweight and obesity in the population with CHD [14]. Therefore, this systematic review elaborates on the prevalence and longitudinal development of overweight and obesity in children and adults with CHD. 

## 2. Materials and Methods

### 2.1. Search Strategy 

A systematic literature search was conducted in the electronic databases PubMed, Cochrane, and Scopus that referred to articles from January 2010 to December 2020. Relevant observational studies in the English language were identified by two independent reviewers. A standard protocol with search terms was developed according to the population, intervention, comparison, outcome, and context (PICO–C) [15] method and applied in the following combination:

1. “congenital heart disease” OR “congenital heart defect” AND
2. “overweight” OR “obesity” OR “adiposity” OR “body constitution”

Medical Subject Headings terms and filters (published in the previous 10 years, humans, preschool child: 2–5 years, child: 6–12 years, adolescent: 13–18 years, adult: 19+ years) were used and appropriately adapted if necessary. 

### 2.2. Data Collection 

Two reviewers screened the relevant articles for title and abstract that had to fulfill the basic inclusion criteria: diagnosed CHD and the measurement of overweight and obesity. At least one of the reviewers had to consider a reference eligible; in case of disagreement, a third reviewer was consulted for a majority decision before full-text analysis. 

Documenting critical appraisal of the included literature, reviewers used the “Study Quality Assessment Tool for Observational Cohort and Cross-Sectional Studies” of the National Heart, Lung and Blood Institute (NHLBI), consisting of a 14-item list assessing potential risk for bias. Accordingly, included studies were categorized as good, fair, or poor [16].

## 3. Results

### 3.1. Study Inclusion 

**Figure 1:**
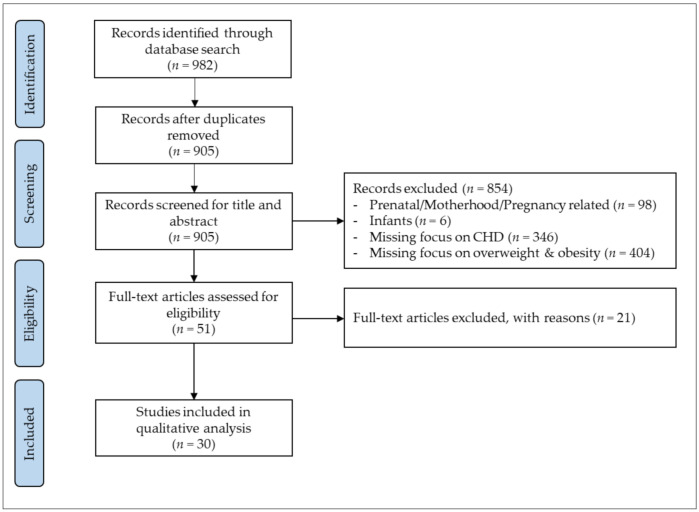
Search and selection process for systematic review according to Preferred Reporting Items for Systematic Reviews and Meta-Analyses (PRISAM). Congenital heart disease (CHD).

In total, 982 potential studies were identified in the initial search, of which 905 remained after duplicates had been removed. After screening titles and abstracts, 51 potential studies were retrieved for full-text analysis, of which 21 studies were excluded due to: main focus on the impact of overweight and obesity (*n* = 12), lacking focus on overweight and obesity (*n* = 5), focus on underweight (*n* = 1) and not exclusively assessing patients with CHD (*n* = 3). Finally, 30 studies with a total of 21,610 CHD patients met the inclusion criteria for this systematic review. The search algorithm and selection process is displayed in Figure 1. 

### 3.2. Study Characteristics 

Of the included studies, 15 studies evaluated 5680 pediatric patients with CHD (range 32–1080 patients) aged 2–18 years, 9 studies evaluated 6657 ACHD (range 54–2424 patients) aged 18–71 years, and 6 studies examined 9273 both pediatric and ACHD (range 50–4496 patients) aged 2–48 years. 

Twenty-six of the included studies were cross-sectional studies that examined the prevalence of overweight and obesity in patients with CHD [17,18,19,20,21,22,23,24,25,26,27,28,29,30,31,32,33,34,35,36,37,38,39,40,41,42], and four were cohort studies that investigated the longitudinal change of the prevalence across the lifespan [10,43,44,45]. The body composition of patients with CHD was compared to a healthy reference cohort in 13 of the included studies [20,21,22,23,24,28,29,30,33,35,40,41,45], whereas 9 studies merely investigated the prevalence of overweight and obesity in patients with CHD [10,17,18,19,24,32,36,38,39]. Table 1 provides further information on the study characteristics and outcomes of the included studies. 

### 3.3. Measuring Overweight & Obesity 

All included studies assessed overweight and obesity with the body mass index (BMI), while two studies additionally performed dual x-ray absorptiometry [18,20], one study performed impedance measurement [35], and one study assessed waist circumference [20]. Based on the BMI, patients were classified as underweight (<18.5 kg/m^2^), normal weight (18.5–24.9 kg/m^2^), overweight (25–29.9 kg/m^2^), obese (≥30 kg/m^2^) and severe obese (≥35 kg/m^2^). BMI values were converted into age and gender-specific z-Scores in a subset of studies [10,17,18,38,40]. Fourteen studies converted the BMI into percentiles based on various growth charts, classifying patients with BMI ≥85th–<95th percentile as overweight and those with BMI ≥ 95th percentile as obese [10,19,23,24,32,33,34,36,38,39,40,41,43,45]. 

### 3.4. Study Quality

According to the NHLBI study quality assessment tool [16], 15 studies received the quality rating “good”, while 9 studies were rated as “fair”. Six studies showed a substantial risk of bias and were rated as “poor”. Various contributing factors have an impact on weight status and should therefore be considered when investigating the prevalence of overweight and obesity. The included studies in this review controlled for age [21,24,35,37,39], age and lesion severity [27], age and sex [10,17,18,20,23,28,31,33,36,38,40,41,43,44], race/ethnicity [29,45], family history, parent’s nutritional status [19], marital status, educational level, and geographic region [34]. Six studies did not take confounders into account and were therefore rated as “poor” [8,22,25,26,37,42]. Comprehensive information on the quality rating can be found in Table 2.

### 3.5. Overweight and Obesity in Children with CHD 

In children with CHD, the prevalence of overweight ranged from 9.5% [36] to 31.5% [39] and the prevalence of obesity ranged from 9.5% [19] to 26.2% [36]. Studies comparing children with CHD to healthy controls reported similar overweight and obesity prevalence in both groups [18,20,21,24,33,35,40,41,45], whereby controls were age-matched [21,35], age and sex matched [20], and age, sex and race/ethnicity matched [45]. 

One study reported decreased BMI in CHD children [23], whereas another study reported increased obesity rates in CHD children compared to healthy controls [33]. CHD participants had a significantly greater waist circumference than controls when controlling for sex, birth weight, physical activity score, and total lean mass [20]. 

Overweight and obesity occurred more frequently in CHD boys than in girls [19,23]. There was no association between overweight and obesity prevalence and CHD severity or surgical status in CHD children [26,40]. Children with cyanotic CHD had a lower prevalence of overweight and obesity than those with non-cyanotic CHD [19,23]. Significantly lower BMI values were found in children with tetralogy of Fallot five years after surgery compared to age-matched controls [21]. Patients with Transposition of the Great Arteries and Coarctation of the Aorta showed significantly higher BMI compared with norm data and other cardiac diagnoses [38,44]. Studies on Fontan children reported a lower prevalence of overweight and obesity at the time of Fontan procedure, which increased to 30% five years post-surgery [10], but showed no significant difference to healthy controls [35]. 

### 3.6. Overweight and Obesity in Adults with CHD 

In adults with CHD, overweight prevalence was found in a range from 22% [24] to 53% [30], while obesity was reported in a range between 7% [22] and 26% [29]. Studies comparing overweight and obesity prevalence with healthy controls found either similar overweight and obesity rates [22,29,43] or lower prevalence compared to the reference cohort [31,42]. However, ACHD showed a decreased prevalence of morbid obesity compared to matched controls [29].

Two studies found no association between CHD severity and prevalence of overweight and obesity [26,29], whereas two other studies reported a reduced prevalence in the more complex classes like total cavopulmonary connection compared to the reference population [31,42]. One study on ACHD reported men being more overweight than women [31], while another study found no sex differences [34]. Hispanic patients were more likely to be obese than white ACHD after adjusting for age and gender [29]. Non-Hispanic Blacks with CHD had a 58% increased risk of obesity in young adulthood and 33% in late adulthood compared to white CHD [28].

BMI was positively associated with impaired NYHA class and cardiovascular medication intake [42]. Metabolic syndrome was more common in ACHD patients than in controls, and obese ACHD patients were more likely to have metabolic syndrome than obese controls [43].

### 3.7. Longitudinal Development of Overweight and Obesity 

The number of overweight and obese children increased during adolescence [27,44]. Also, in adults with CHD, age correlated with increased BMI [29,31,34,42], with similar results found in the general population [29,31]. Generally, adults had significantly higher BMI scores and were more likely to be overweight or obese compared with younger samples [26]. The odds of being overweight or obese as an adult were found to be three times higher if their childhood BMI was already above the 85th percentile [24].

## 4. Discussion

This systematic review shows that the overall distribution of overweight and obesity is consistent with the prevalence in the general population, in which overweight and obesity rates have increased dramatically in recent decades [37]. Particularly, CHD patients with increasing age, male gender, and Hispanic origin are at risk for overweight and adiposity. These findings are worrisome, as the CHD population is at higher risk of developing metabolic syndrome and premature morbidity and mortality compared to their healthy peers [43]. Obesity is associated with an increased risk of cardiovascular diseases, like heart failure and coronary artery disease [46]. Excessive body adipose tissue impacts the vessel wall by changes in blood pressure, glucose level, lipid metabolism, and systemic inflammation [47]. In children and adults with CHD, overweight and obesity were shown to be associated with cardiac comorbidities, increased cardiac medication intake, and higher systolic and diastolic blood pressure compared to normal-weight patients. According to this recently published study, the elevated risk of morbidity and mortality in the CHD population is exacerbated by obesity [27]. Obesity has been associated with adverse perioperative outcomes [48] as well as adverse short-term outcomes after cardiothoracic surgery in patients with CHD [49]. In contrast, another study on ACHD reported that overweight and moderate obesity were associated with reduced mortality rates, especially in symptomatic ACHD patients and those with complex underlying cardiac defects, replicating the so-called “obesity paradox” in the general population [14]. It was also shown that overweight and obesity are associated with lower heart failure rates in ACHD [24]. In the elderly, this may indeed be true, as a slightly elevated weight above normal BMI can be a resource to build upon when facing surgical procedures or beginning aggressive drug therapy. However, overweight and obesity are among the most significant contributors to illness and adverse health outcomes and should be prevented or reduced at all costs, at least in younger patients with CHD. 

Underlying causes and effects of the high overweight and obesity prevalence in the CHD population are multifactorial. Heart failure-associated reasons such as medication intake were identified along with the already mentioned behavioral factors. Two major factors influencing the CHD population are physical inactivity due to restrictions or overprotection by physicians and parents, as well as weight gain interventions in infancy, which can develop into excess weight later in life [17]. Part of the included studies examined the association between overweight and obesity and contributing factors. Yang et al. reported an association between sedentary behavior and increased overweight and obesity prevalence in CHD children [41]. BMI has been shown to be negatively associated with exercise duration [32] and exercise capacity in the CHD population [42].

Continuous monitoring of weight status and interventions to reduce the prevalence of overweight and obesity are required in this patient cohort at high risk for acquired cardiovascular disease [37]. Interventions such as lifestyle and nutritional counseling to reduce risk factors for obesity should begin in early childhood and include parental education. 

### Limitations and Further Research 

The classification of overweight and obesity in the majority of included studies refers to the BMI, which, however, does not analyze fat distribution and body composition. Abdominal fat, in particular, has been associated with various pathologies [45]. Therefore, further studies should specifically address abdominal fat in order to quantify the visceral component of adipose tissue [31]. Moreover, BMI is based on both fat mass and lean mass and therefore carries the risk of classifying muscular patients as overweight [37]. The majority of included studies have not considered race and ethnicity as potential confounders, even though their influence on weight status is well established [44]. 

## 5. Conclusions

The prevalence of overweight and obesity in children and adults with CHD reflects that of the general population, demonstrating that the growing obesity pandemic is also affecting the CHD population. Such a high prevalence of overweight and obesity is particularly worrying in the context of CHD. We hope that these results will raise awareness of this issue and encourage appropriate health-promoting interventions and individual consultations.

## Figures and Tables

**Table 1:** Study characteristics and outcomes.

Study	CHD, *n* (♀)	Control Group, *n* (♀)	CHD Diagnosis or Severity (*n*)	Age ± SD (Range), y	Reference	Body Constitution	Prevalence (%) Overweight/Obesity (Both)	Results
PEDIATRIC CHD (*n* = 15)								
Tamayo et al., 2015 [44]	725 (319)	-	ASD (116), VSD (152), AVSD (116), TGA (138), UVH (73), ToF/DORV (130)	Median age at complete repair 0.6	Center of Disease Control Atlanta	*	28/17	Proportion of overweight & obesity increased over time (*p* = 0.02). Patients with TGA showed higher BMI at any time point compared with other cardiac diagnoses (*p* < 0.001).
Wellnitz et al., 2015 [10]	84 (40)	-	undergoing Fontan palliation	At Fontan surgery: 4.72 (IQR: 3.51–5.14)	Centers for Disease Control	*	(Fontan: 10.7 1 y after: 20.3 5 y after: 30)	Time of Fontan: lower percentage of overweight/obesity compared with US children (10.7% versus 30.4%). 1 y after Fontan: overweight/obese children increased to 20.3%. 5 y after Fontan: increased to 30%. Increase in BMI after Fontan significantly associated with Hispanic ethnicity (*p* < 0.001).
Powell et al., 2020 [35]	47 (22)	165 (86) age-matched	Fontan circulation	15.0 ± 3.1	-	BIA	23/-	Patients with Fontan had similar BMI as normal controls, but had higher body fat percentage (*p* = 0.03), lower lean muscle mass (*p* = 0.005) and skeletal muscle mass (*p* = 0.004).
O’Byrne et al., 2018 [32]	253 (106)	-	ToF (78), TGA (20), Fontan (74)	13.1 ± 2.9	United States Centers for Disease Control	*	15/11	Increasing exercise duration was associated with lower BMI (*p* = 0.01). Restriction to mild exertion and participation in low-intensity exercise were both associated with increased BMI.
Aguilar et al., 2015 [17]	551 (251)	-	CoA (79), VSD (281), ToF (66), TGA (65), SV (34), HLHS (26)	Median 7.5	Center for Disease Control & Prevention Growth Chart	-		2–7 y: BMI Z-score increased during early childhood in VSD, and ♀ patients with CoA or HLHS. 8–15 y: BMI Z-score increased in those with CoA and ToF. 2–20 y: BMI Z-score gain between 2–20 y was increased in CoA (♀ only), HLHS (♀ only) VSD, and decreased in SV (♀only) and TGA.
Barbour-Tuck et al., 2020 [20]	32 (12)	23 (10) age- and sex matched	Fontan (7), ToF (5), HT (4), TGA (3), ASD (2), PS (2), VSD (1), CoA (2), AS (1), TA (1), CM (2), DORF (2)	10.9 ± 2.6	-	WC & DXA	Mean BMI 18.9 ± 4.7 kg/m^2^	CHD participants had a significantly greater waist circumference than controls when controlling for sex, birth weight, physical activity, and total lean mass. CHD and control groups were similar in BMI, total fat mass, total lean mass, percent fat mass, percent lean mass.
Welisch et al., 2013 [40]	1080 (483)	1083 (472)	VSD, ASD, PDA (146); Fallot, TGA, TAC, VD, AVSD, CoA (369); VD (271), F&M&S (40), Shunt (227), other (27)	9.0 ± 4.7	Center for Disease Control and Prevention BMI curve	*	(18.2)	No significant difference concerning weight category between CHD and healthy controls. No difference in overweight/obesity prevalence between operated and non-operated CHD. Age and gender not risk factors for being overweight/obese.
Barbiero et al., 2014 [19]	316 (140)	-	VSD (76), ASD (61), ToF (43), PA (6), others (130)	2–5 y: 67 6–11 y: 138 12–18 y: 111	according to the WHO-2006/07	*	17.4/9.5	Excess weight was more common among ♂ (60%). Family history of obesity was associated with excess weight (*p* = 0.001). In patients with cyanotic lesions, overweight was less frequent than in acyanotic (23.3% vs. 27.7%).
Briston et al., 2017 [21]	137 (66)	1:1 age-matched controls	ToF	NR	-	**	19/19	In the first 5 y of age and in the first 5 y postoperatively, the ToF cohort had a significantly lower BMI compared with the control group (*p* = 0.042 and *p* = 0.028). Afterward, no sig. difference between CHD & healthy controls (*p* = 0.079).
Chen et al., 2012 [23]	Child: 705 (368) Adolescent: 219 (192)	Child: 18,753 (7798) Adolescent: 15,014 (7666)	VSD (319), ASD II (209), PVD (99), PDA (72), AVD (38), CAVF (15), ToF (65) ECD (17), TGA (14), VSD with CoA (13), CoA (12), VSD with R (12), PA (7), EBS (8), Other (22)	Child: 6.4 ± 0.5 Adolescent: 15.5 ± 0.6	Nutrition and Health Survey in Taiwan	*	(Child: 14.5, Adolescent: 26.5%)	The prevalence of overweight/obesity in CHD adolescents close to controls. In ♀ fewer CHD children were overweight/obese (12.2% vs. 18.7%, *p* = 0.002). ♂ with moderate to severe CHD had a lower prevalence of overweight/obesity (*p* = 0.025). Children with cyanotic CHD had significantly lower prevalence of overweight/ obesity (1.5% vs. 15.5%, *p* = 0.003) than those with non-cyanotic.
Perin et al., 2019 [33]	220 (95)	220 (93)	No residual defect (142), residual defects (58), UVH (20)	11.4 ± 2.8	2007 World Health Organization growth charts	*	(35.4)	Higher prevalence of obesity in CHD patients (22.7%) compared to 15.5% in healthy subjects (*p* = 0.015). Higher proportion of obese children in the age 6–11 subgroup (28.6%) compared to the age 12–17 subgroup (16%, *p* = 0.006).
Ray et al., 2011 [36]	84 (33)	-	Mild (21.5%), Moderate (16.7%), Surgically (40.5%), Complex (21.4%)	12 ± 1.4	Centers for Disease Control and Prevention	*	9.5/26.2	% of children who were overweight/obese ranged from 22% to 44%, with the lowest incidence in those with moderate disease or that were surgically corrected and the highest incidence in those with mild disease.
Steele et al., 2019 [39]	968 (419)	-	Cyanotic (232), Repaired or palliated (719), Acyanotic (217), Electrophysiologic (184)	13.3 (8.8–16.4)	Center for Disease Control	*	31.5/16.4	Children with overweight/obesity were older (*p* < 0.001), had lower median household income (*p* = 0.031), and more often complex CHD (*p* = 0.008). Children with CHD have an increased risk of becoming overweight & obese in early childhood.
Yang et al., 2020 [41]	97 (45)	-	ASD (33), VSD (30), PDA (9), TGA (9), ToF (12), Endocardial cushion defect (4)	9.7 ± 1.5	50th-percentile BMI for Taiwanese children	*	(14.4)	BMI did not differ between CHD and children in the general population. Greater obesity in children with mild heart disease (*p* = 0.04). Sedentary behaviors, cardiomegaly, and the NYHA class II–IV were associated with being overweight/obese.
Smith-Parris et al., 2014 [38]	160 (59)	Adult with AS, PS or ASD	Underwent CoA repair	median age at follow-up of 14 y (range, 4.6–36.7 y)	National Health & Nutrition Examination Survey	*	(47)	At age 5 y, patients with CoA had significantly greater BMI z-scores compared with age-sex matched normal data (*p* < 0.001). The proportion of excess weight in COA significantly increased over time (*p* < 0.001). Adults with repaired COA developed obesity at a greater rate than those with either AS (*p* = 0.004) or with PS or ASD (*p* < 0.001).
CHILDREN & ADULTS (*n* = 6)								
Chung et al., 2016 [24]	Child: 395 Adult: 129 (58)	-	Fontan circulation	Child: 2–5 y: 401 6–11 y: 333 12–19 y: 217 Adult: 27.8 ± 6.8	Center for Disease Control	*	Adults: 22/17 (Children: 15)	The likelihood of being overweight/obese as an adult was three times higher if there was a BMI ≥ 85th percentile in childhood (*p* < 0.01). Pediatric rates of overweight/obesity comparable to healthy controls. No race or gender differences between overweight/ obese. Overweight/obesity in adulthood was associated with lower heart failure rates (4 vs.19%, *p* = 0.03).
Avitabile et al., 2014 [18]	50 (24)	992 healthy controls	Fontan Median 9.3 years from Fontan	Median: 11.5 (5.1–33.5)	2000 National Center for Health growth statistics	DXA	BMI z-Score: 0.15 ± 0.98	BMI Z-scores did not differ between Fontan and healthy controls (0.15 ±0.98 vs 0.35 ± 1.02, *p* = 0.18). Whole-body lean mass Z-scores were lower in the Fontan participants compared with reference (*p* = 0.003).
Jackson et al., 2019 [28]	4496 (2158)	-	36% simple, 50% moderate, 14% complex	6–12 y: 1327 13–18 y:1005 19–39 y:1312 40+ y: 842	Centers for Disease Control and Prevention	**	White&Black: 6–12 y: 15/19 & 18/15 13–18 y: 18/20 & 21/27 19–39 y: 31/27 & 28/42 40+: 34/40 & 32/52	White children with CHD had a higher prevalence of obesity (18.6%) compared to healthy controls (13.8%) (*p* < 0.01). White young adults with CHD had a lower prevalence of obesity (27.4%) as compared with white young controls (31.1%) (*p* < 0.01). No differences between white CHD & healthy adolescents (19.8% vs. 20.8%), as well as black CHD survivors of all ages. Blacks with CHD had a 58% increased risk of obesity in young adulthood and 33% in late adulthood.
Harris et al., 2018 [26]	Youth: 88 (36) Adult: 102 (47)	-	Youth: 32% mild, 40% moderate, 28% complex Adult: 30% mild, 47% moderate, 23% complex	Youth: 17.2 ± 1.1 Adults: 35.4 ± 12.9	International Obesity Task Force criteria	**	Youth: 10/11 Adults: 30/22	More adults than youth overweight/obese (52% vs. 22%, *p* < 0.001). Group mean BMI and prevalence of weight categories were not different by sex in adults, but in youth, more ♀ than ♂ were overweight/ obese (33% vs. 13%, *p* = 0.026).
Weinreb et al., 2019 [45]	223 (97)	223 1:1 age, sex & race matched controls	34% simple, 32% moderate, 34% complex	5–11 y 95 11–15 y: 64 16–20 y: 64	Commission for Disease Control BMI	*	(25)	Mean BMI% did not differ between CHD sample and paired controls over a 5 y period. Significant increased BMI% change in the age cohort of 5–10 y (*p* = 0.04), in ♂ sex (*p* = 0.01) and status-post surgery (*p* = 0.02).
Jackson et al., 2020 [27]	3790 (1868)	-	36% simple, 50% moderate, 14% severe	6–18 y: 1927 19–39 y: 1139 40+ y: 724	Center for Disease Control & Prevention	*	Youth: 17/18 Young adults: 30/30 Adult: 33/40	The proportion of individuals with overweight/obesity increased with age (*p* < 0.001). A higher proportion of individuals with moderate lesion severity (29%) had obesity compared to simple (24%) and complex (18%, *p* < 0.001) lesion severity
ADULTS (*n* = 9)								
Lerman et al., 2017 [29]	1451 (719)	1451 (719) age, gender & race-matched	Simple (1007), Complex (299), Unclassified (145)	52 ± 20	-	**	33.5/25.6	ACHD were equally as likely to be overweight/obese as controls; ACHD decreased the prevalence of morbid obesity. Age correlated with increased BMI in ACHD and controls (*p* < 0.001). BMI was similar across all disease severity groupings. Hispanic patients were more likely to be obese than white patients (*p* = 0.02) & controls (*p* = 0.01).
Buys et al., 2013 [22]	103 (33)	-	CoA	28.7 ± 6.3	Belgian health survey	**	♂: 27/7 ♀:18/12	Weight status was similar to the overall Belgian population, with a tendency toward higher BMI. A tendency towards higher incidence in obesity in ♀ patients.
Lui et al., 2017 [8]	178 (87)	-	Fallot (26%), TGA (20%), Fontan with UVH (15%)	37.1 ± 12.6	-	**	53/21	Excess adiposity was the most common risk factor for developing atherosclerotic cardiovascular disease in ACHD.
Pike et al., 2012 [34]	54 (28)	66 age, sex, ethnicity, family, region & education	Fontan	26 ± 9	Center for Disease Control	*	(21)	No sex differences in BMI in the SVCHD group. Patients >21 y higher BMI compared with patients ≤ 21 years of age (*p* = 0.01).
Fedchenko et al., 2019 [25]	72 (30)	-	CoA	median 43.5 (20–71)	-	**	38.9/9.7	Cardiovascular risk factors were prevalent among patients with CoA.
Zaqout et al., 2019 [42]	539 (248)	1737 (896) from Belgium	VSD (78), ASD (54), VPS (30), PDA (14), MVD (9), AS/AR (86), CoA/VSD (73), TOF (74), AVSD (34), TGA (14), TGA/ ASO (16), TCPC (30), TrA (6), PA/DORV (21)	32.0 ± 9.3	-	**	23.7/10	ACHD patients had lower BMI than healthy controls (*p* = 0.012). BMI was positively associated with age (*p* < 0.001). Men in the mild & severe group (*p* = 0.007; *p* = 0.023) and women in the severe group (*p* < 0.001) had lower BMI compared to the reference group. Men with VSD, CoA and Fontan and women with Fontan had lower BMI than controls.
Deen et al., 2016 [43]	448 (230)	448 sex & age matched	ToF (95), VD (86), AAA (77), TGA (51), Fontan (43), VSD (21), AVSD (21), ccTGA (20), APVR (20), EBS (16), ASD (11), CAA (8), TAC (7), EM (8), PA (8), other (51)	32.4 ± 11.3	International Diabetes Foundation Criteria	**	-/16.1	The obesity rate was similar between matched ACHD and healthy controls. Metabolic syndrome was more common in ACHD patients than in controls (15.0% versus 7.4%). Obese ACHD patients were more likely to have metabolic syndrome than obese controls (93.1% vs. 44%).
Sandberg et al., 2015 [37]	2424 (1021)	4605 age-stratified	Fontan (97), AS (122), ToF (238), PA/DORV (81), CoA (414), VSD (497), ASD (414), AS/AR (561)	18–50 y	-	**	Simple: 24.0 ± 4.6 kg/m^2^ Complex: 22.6 ± 4.2 kg/m^2^	Men with PA/DORV, AS/AR, and Fontan/TCPC had a lower prevalence of overweight/obesity (*p* < 0.001) than healthy controls. No differences in BMI in CoA, VSD, ASD, or AS/AR and intervention vs. no intervention. Complex lesions: age & cardiovascular medication associated with a lower BMI. Simple lesion: age, impaired NYHA class, and cardiovascular medication associated with higher BMI.
Malavazos et al., 2019 [31]	1388 (776)	145,992 sex & age-stratified	Septal heart defects & left-to-right shunt (864), conotruncal heart disease (209), valve defects & aortic defects (247), UVH (68)	41.5 ± 13.2	-	**	26.7/9.6	Lower prevalence of overweight in ACHD (27%) and in great complexity class (16%) compared to Italian reference (32%). In great complexity class, the prevalence of obesity was significantly lower (3.1%). Men were more likely to be overweight than women in ACHD population (34.64% vs. 20.49%). Overweight/Obesity increased with age.

AAA, aortic arch anomalies; APVR, Anomalous pulmonary venous return; AS, aortic valve stenosis; AS/AR, aortic regurgitation or Ross procedure; ASD, atrial septal defect; ASO, arterial switch operation; AVD, aortic valve disease; AVSD, atrioventricular septal defect; BIA, bioelectrical impedance analysis; BMI, body mass index; CAA, coronary artery anomaly; CAVF, Coronary arterio-venous fistula; CHD, congenital heart disease; CM, cardiomyophathy; CoA, coarctation of the aorta; DORV, double outlet right ventricle; DXA, Dual energy X-ray absorptiometry; EBS; Ebstein; EM, Eisenmenger; F&M&S, Fontan & Mustard & Senning; HLHS, hypoplastic left heart syndrome; HT, heart transplantation; IQR, interquartile range; MVP, mitral valve disease; NR, not reported; PA, pulmonary atresia; PDA, patent ductus arteriosus; perc., percentile; PS, pulmonary valve stenosis; PVD, pulmonary valve disease; R, regurgitation; SD, standard deviation; SV, single ventricle physiology; TA, tricuspid atresia; TGA, transposition of the great arteries; ToF, tetralogy of Fallot; TrA, truncus arteriosus; UVH, uni-ventricular heart; VD, valvar disease; VPS, valvular pulmonary stenosis; VSD, ventricular septal defect; WC, waist circumference; y, year; *: Overweight: BMI ≥ 85th–95th perc.; Obesity: BMI ≥ 95th perc.; **: Overweight: BMI 25–29.9 kg/m^2^; Obesity: BMI ≥ 30 kg/m^2^; Morbidly obese: BMI ≥ 40 kg/m^2.^

**Table 2:** Quality assessment according to the NHLBI Quality Assessment Tool for Observational Cohort and Cross-Sectional Studies.

Study	Type	Q1	Q2	Q3	Q4	Q5	Q6	Q7	Q8	Q9	Q10	Q11	Q12	Q13	Q14	Quality
Pediatric CHD (*n* = 15)																
Barbour-Tuck et al., 2020 [20]	CSS	✔	✔	CD	-	-	-	-	✔	-	-	✔	-	NA	✔	Fair
Powell et al., 2020 [35]	CSS	✔	✔	✔	✔	-	-	-	✔	✔	-	✔	-	NA	✔	Good
Yang et al., 2020 [41]	CSS	✔	✔	-	✔	-	-	-	✔	✔	-	✔	-	NA	✔	Fair
Perin et al., 2019 [33]	CSS	✔	✔	✔	-	-	-	-	✔	✔	-	✔	-	NA	✔	Fair
Steele et al., 2019 [39]	CSS	✔	✔	✔	✔	-	-	-	✔	✔	✔	✔	-	NA	✔	Good
O’Byrne et al., 2018 [32]	CSS	✔	✔	✔	✔	✔	-	-	-	✔	-	✔	-	NA	✔	Fair
Briston et al., 2017 [21]	CSS	✔	✔	CD	✔	-	-	-	✔	✔	-	✔	-	NA	✔	Good
Aguilar et al., 2015 [17]	CSS	✔	✔	✔	✔	-	-	-	✔	✔	✔	✔	-	NA	✔	Good
Tamayo et al., 2015 [44]	CS	✔	✔	CD	✔	-	✔	✔	✔	✔	✔	✔	-	NA	✔	Good
Wellniz et al., 2015 [10]	CS	✔	✔	-	✔	-	✔	✔	-	✔	✔	✔	-	NR	✔	Fair
Barbiero et al., 2014 [19]	CSS	✔	✔	CD	✔	✔	-	-	-	✔	-	✔	-	NA	✔	Fair
Smith-Parrish et al., 2014 [38]	CSS	✔	✔	✔	✔	-	-	-	✔	✔	✔	✔	-	NA	✔	Good
Welisch et al., 2013 [40]	CSS	✔	✔	✔	✔	-	-	-	✔	✔	NA	✔	-	NA	✔	Good
Chen et al., 2012 [23]	CSS	✔	✔	✔	✔	-	-	-	✔	✔	-	✔	-	NA	✔	Good
Ray et al., 2011 [36]	CSS	✔	✔	✔	✔	-	-	-	✔	✔	-	✔	-	NA	✔	Fair
Pediatric & ACHD (*n* = 6)																
Jackson et al., 2020 [27]	CSS	✔	-	CD	✔	-	-	-	-	✔	NA	✔	-	NA	✔	Fair
Jackson et al., 2019 [28]	CSS	✔	✔	✔	✔	-	-	-	✔	✔	-	✔	-	NA	✔	Good
Weinreb et al., 2019 [45]	CS	✔	✔	✔	✔	-	✔	✔	✔	✔	✔	✔	-	NA	✔	Good
Harris et al., 2018 [26]	CSS	✔	✔	CD	✔	-	-	-	✔	✔	NA	✔	-	NA	-	Poor
Chung et al., 2016 [24]	CSS	✔	✔	CD	✔	-	-	-	✔	✔	✔	✔	-	NA	✔	Good
Avitabile et al., 2014 [18]	CSS	✔	✔	CD	✔	-	-	-	✔	✔	-	✔	-	NA	✔	Fair
ACHD (*n* = 9)																
Fedchenko et al., 2019 [25]	CSS	✔	✔	-	✔	-	-	-	-	✔	-	✔	-	NA	-	Poor
Malavazoz et al., 2019 [31]	CSS	✔	✔	CD	✔	-	-	-	✔	✔	NA	✔	-	NA	✔	Good
Zaqout et al., 2019 [42]	CSS	✔	✔	✔	-	-	-	-	✔	✔	-	✔	-	NA	-	Poor
Lerman et al., 2017 [29]	CSS	✔	✔	✔	✔	-	-	-	✔	✔	NA	✔	-	NA	✔	Good
Lui et al., 2017 [8]	CSS	✔	✔	✔	✔	-	-	-	✔	✔	-	✔	-	NA	-	Poor
Deen et al., 2016 [43]	CS	✔	✔	✔	-	-	✔	NA	✔	✔	NA	✔	-	NA	✔	Good
Sandberg et al., 2015 [37]	CSS	✔	✔	✔	✔	-	-	-	✔	✔	NA	✔	-	NA	-	Poor
Buys et al., 2013 [22]	CSS	✔	✔	✔	✔	-	-	-	NA	✔	NA	✔	-	✔	-	Poor
Pike et al., 2012 [34]	CSS	✔	✔	CD	✔	-	-	-	✔	✔	-	✔	-	NA	✔	Good

ACHD: adults with congenital heart disease, CD: cannot determine, CHD: congenital heart disease, CS: cohort study, CSS: cross-sectional study, NA: not applicable, NR: not reported, ✔ denotes “Yes”, - denotes “No”.
